# The Public Health Response and Epidemiologic Investigation Related to the Opening of a *Bacillus anthracis–*Containing Envelope, Capitol Hill, Washington, D.C.

**DOI:** 10.3201/eid0810.020332

**Published:** 2002-10

**Authors:** Vincent P. Hsu, Susan L. Lukacs, Thomas Handzel, James Hayslett, Scott Harper, Thomas Hales, Vera A. Semenova, Sandra Romero-Steiner, Cheryl Elie, Conrad P. Quinn, Rima Khabbaz, Ali S. Khan, Gregory Martin, John Eisold, Anne Schuchat, Rana A. Hajjeh

**Affiliations:** * Centers for Disease Control and Prevention, Atlanta, Georgia, USA; † National Naval Medical Center, Bethesda, Maryland, USA; ‡ Office of the Attending Physician, U.S. Capitol, Washington, D.C., USA

**Keywords:** *Bacillus anthracis*, nasal swabs, epidemiology, bioterrorism, postexposure prophylaxis

## Abstract

On October 15, 2001, a U.S. Senate staff member opened an envelope containing *Bacillus anthracis* spores. Chemoprophylaxis was promptly initiated and nasal swabs obtained for all persons in the immediate area. An epidemiologic investigation was conducted to define exposure areas and identify persons who should receive prolonged chemoprophylaxis, based on their exposure risk. Persons immediately exposed to *B. anthracis* spores were interviewed; records were reviewed to identify additional persons in this area. Persons with positive nasal swabs had repeat swabs and serial serologic evaluation to measure antibodies to *B. anthracis* protective antigen (anti-PA). A total of 625 persons were identified as requiring prolonged chemoprophylaxis; 28 had positive nasal swabs. Repeat nasal swabs were negative at 7 days; none had developed anti-PA antibodies by 42 days after exposure. Early nasal swab testing is a useful epidemiologic tool to assess risk of exposure to aerosolized *B. anthracis.* Early, wide chemoprophylaxis may have averted an outbreak of anthrax in this population.

 In the fall of 2001, a series of envelopes containing *Bacillus anthracis* spores were sent via the U.S. Postal Service (USPS) to cities in Florida and New York. Consequently, many persons, including staff on Capitol Hill, received training on how to respond to suspicious envelopes that might contain *B. anthracis* spores. This training was based on previously prepared recommendations for a comprehensive response to biological attacks using *B. anthracis* (1–3). On October 15, 2001, an envelope addressed to Senator Tom Daschle containing *B. anthracis* spores was opened by one of his staff members. While the bioterrorism events in Florida and New York came to the attention of public health authorities only when persons were diagnosed (4–7) with anthrax, the event on Capitol Hill was different—the presence of *B. anthracis* spores was suspected immediately, allowing appropriate response and prompt initiation of chemoprophylaxis in exposed persons. A known source of exposure allowed a rapid epidemiologic investigation, using nasal swab cultures for *B. anthracis*, environmental sampling, and serologic testing. Although previous epidemiologic studies have used nasal swabs and serologic tests to assess *B. anthracis* exposure and subclinical (asymptomatic) infection in endemic and outbreak settings (8–11), the usefulness of these tools in the context of a bioterrorism event is not known.

 We describe here the initial public health response to the opening of the contaminated envelope on Capitol Hill and the epidemiologic methods used to determine the exposed area and the population at risk for developing anthrax. While the public health response later included the letter traceback through the entire postal system, including identification and prophylaxis of at-risk USPS employees (12), we limit our discussion to Capitol Hill. The results and epidemiologic importance of environmental sampling for *B. anthracis*, although briefly mentioned, will be the focus of a separate paper.

## Timeline of Events

 On October 15, 2001, at 9:45 a.m., a staff member on the 6th floor of the Hart Senate Office Building (HSOB) in the office of Senate Majority Leader Tom Daschle cut open a taped business envelope containing a letter and a powdery substance (Table 1). Upon noticing a burst of dust, she placed the letter on the floor and notified the U.S. Capitol Police. Within 5 minutes of being notified, officers were at the scene. The hazardous device unit of the Capitol Police arrived minutes later. The officers and emergency response personnel, referred to as first responders, arrived with respiratory personal protective equipment (PPE) on hand, but equipment was not put on until after arrival at the scene. These officers tested the powder for *B. anthracis* spores twice, using commercial rapid tests. Preliminary results obtained within 15 minutes suggested that the powder contained *B. anthracis*. Laboratories at the U.S. Army Medical Research Institute of Infectious Diseases (USAMRIID) in Fort Detrick, Maryland, later confirmed these preliminary results.

**Table 1:** Timeline of events within the Hart Senate Office Building, Washington, D.C., October 15, 2001^a^

Time of day	Event /response
9:45 a.m.	Staff person opens letter containing *Bacillus anthracis* spores.
9:55 a.m.	First responders arrive at scene.
10:00 a.m.	Hazardous device unit arrives at scene and performs initial tests for *B. anthracis.*
10:15 a.m.	First rapid test is positive for *B. anthracis.*
10:30 a.m.	Ventilation system turned off. Second rapid test is positive. OAP^b^ begins nasal swab testing and antibiotic chemoprophylaxis distribution.
10:40 a.m.	6th floor staff moved to 9th floor; swabbing continues; staff later moved to 5th floor.
3:00 p.m.	Senators Daschle and Feingold’s staff allowed to go home.

^a^One responder with a positive nasal swab who was in the 6^th^-floor hallway did not enter the Daschle or Feingold suites and was not included in this table. ^b^OAP, Office of the Attending Physician.

 At approximately 10:30 a.m., the ventilation system was shut off. Medical staff from the Office of the Attending Physician (OAP), U.S. Capitol, began collecting nasal swabs for *B. anthracis* culture from staff members in Senator Daschle’s office, from staff in an adjacent office belonging to Senator Russell Feingold, and from the first responders; in addition, an initial 3-day antimicrobial postexposure prophylaxis regimen consisting of ciprofloxacin, 500 mg twice a day, was given to these persons. Only the person who opened the contaminated envelope removed and changed her clothing and was decontaminated with soap and water. All others washed their hands with soap and water.

 Next, first responders led employees from the two 6th-floor offices to the 9th floor of the building, where further samples were taken from nares and clothing. After testing, these employees were led back to Senator Daschle’s 5th-floor office, where other staff members were detained. At approximately 3:00 p.m., the staff members were allowed to go home.

 Employees in other offices continued working until the close of business. The southwest quadrant of the building was closed the morning of October 16, and a decision was made to close the entire HSOB that evening. During the next 3 days, OAP continued to collect nasal swabs for *B. anthracis* for all HSOB employees present on October 15 and for others on Capitol Hill who requested these tests. OAP also gave those tested an initial 3-day antimicrobial prophylaxis, pending final confirmation of the presence of *B. anthracis* spores and results of the epidemiologic investigation.

## Methods

 A team from the Centers for Disease Control and Prevention (CDC) arrived in Washington D.C., on the morning of October 16 to begin the epidemiologic investigation. To identify the group of persons who needed prolonged antimicrobial prophylaxis on the basis of likely exposure to *B. anthracis* spores, we sought to define an exposure area of higher risk.

 To identify other facilities that may have been contaminated with *B. anthracis* spores, the contaminated envelope was traced back through the congressional mail distribution system before its arrival in Senator Daschle’s office. To define the exposure area for HSOB, we obtained floor diagrams for the 5th and 6th floors and information about the ventilation system from the Office of the Architect of the Capitol, which maintains and operates the U.S. Capitol complex. Multiple environmental samples were taken from these facilities by a variety of techniques (13).

 The population at risk of developing anthrax was defined as persons in the exposed area during or after the time the contaminated envelope was processed or opened. To identify each person who may have been within the exposure area, employee lists were obtained from staff managers for each affected facility; in HSOB, managers for individual senators’ offices within the defined exposure area were contacted to obtain employee and visitor lists. We identified responders within HSOB, such as law enforcement and medical personnel, by contacting supervisors for a comprehensive list of those who were in the area. To identify other visitors or nonemployees, press conferences were used to relay the appropriate information.

 Within 9 hours of the initial event, nasal swab specimens were collected for all persons in Senator Daschle’s and Senator Feingold’s offices and for all first responders. As mentioned earlier, further specimens were collected by OAP, for 4 days after the opening of the contaminated envelope, from employees of HSOB and others on Capitol Hill. Specimens were collected with Dacron fiber-tipped sterile swabs and sent for *B. anthracis* culture at the National Naval Medical Center in Bethesda, Maryland. Persons with initial positive nasal swabs for *B. anthracis* had repeat nasal swabs at 7 days postexposure and were administered a questionnaire about symptoms consistent with anthrax disease. In addition, serum specimens were obtained from these persons and tested at the CDC Meningitis and Special Pathogens Laboratory for the presence of immunoglobulin (Ig) G antibodies to *B. anthracis* protective antigen (anti-PA) at 7, 21, and 42 days postexposure.

 In collaboration with OAP, efforts were made to ensure that all exposed persons were contacted and that they received appropriate prophylaxis with ciprofloxacin, or in the cases of persons unable to tolerate a quinolone, with doxycyline. OAP closely monitored persons who came to the clinic with respiratory symptoms; follow-up surveys were later conducted on persons receiving long-term antibiotic prophylaxis.

## Results

### Defining the Exposure Area and Population at Risk

 Within Capitol Hill, the traceback of the contaminated envelope before its arrival in Senator Daschle’s office showed that it had been screened through a mail facility on P Street and then through the Senate nonpublic mailroom, located in the Dirksen Senate Office Building (Table 2). Nasal swabs for *B. anthracis* in employees of both mail facilities were negative; however, since exposure to *B. anthracis* spores may have occurred during mail handling of the contaminated letter, the Dirksen mailroom and the entire P Street facility, which was an open warehouse, were defined as exposed areas. Additionally, positive environmental samples for *B. anthracis* were found in the mailroom in the Ford House Office Building, where mail to the House of Representatives is processed. Although the contaminated envelope did not pass through the Ford Building mailroom, the potential of aerosolization of spores from processing equipment, as well as the possibility of an additional contaminated envelope, warranted its designation as an exposed area.

**Table 2:** Defined exposure areas and identification of persons at risk from *Bacillus anthracis*–containing envelope, Washington, D.C.

Defined exposure area	Environmental samples positive?	No. persons identified at risk	No. positive nasal swabs
SE quadrant, 5th and 6th floors, Hart Senate Office Building	Yes	442	28
P Street mail-processing facility	Yes	62	0
Mailroom, Dirksen Senate Office Building	Yes	40	0
Mailroom, Ford House Office Building	Yes	81	0
Totals		625	28

 Senator Daschle’s suite is located on the 5th and 6th floors of the southeast quadrant, with an open internal staircase joining the floors. An adjacent suite occupied by staff of Senator Feingold has a similar layout. Both adjacent offices share a common hallway that serves as the main entry to the 6th-floor office, but no door connects the Daschle and Feingold suites. A single ventilation system supplies and exhausts air for the nine floors in the southeast quadrant, independently of other areas in the building.

 In HSOB, where the primary release of *B. anthracis* spores occurred, all persons with nasal cultures positive for *B. anthracis* were clustered in and around Senator Daschle’s office and were located on either the 5th or 6th floor (see below). Preliminary environmental sampling results were positive for *B. anthracis* spores from within the same rooms occupied by persons with positive nasal cultures. The location of the contaminated office was within the shared ventilation space of the southeast quadrant of the building. The exposure area in HSOB was thus defined as the southeast quadrant of the 5th and 6th floors. Within these four designated exposure areas (5th- and 6th-floor southeast quadrant, P Street facility, and the Dirksen and Ford Building mailrooms), 625 persons were identified as employees, visitors, or otherwise being within the exposed areas (Table 2). More than 2,000 persons received an initial 3-day course of antibiotics, but only the 625 persons from the defined exposure areas were recommended to receive 60 days of chemoprophylaxis.

### Nasal Swabs Results

 OAP obtained nasal swabs for *B. anthracis* culture from 2,172 persons during October 15–October 18, including the 625 persons identified at risk. Of these, 71 were known to be in the immediate exposure area within the first hour of the event in which the contaminated envelope was opened (Table 3); 65 were Senate staff, and 6 were first responders. A total of 28 persons had positive nasal cultures for *B. anthracis*; all positive results were from specimens obtained on October 15 between 10:30 a.m. and 7:00 p.m. The median age of these persons was 27 years (range 21–57). All persons positive for *B. anthracis* entered either Senator Daschle’s or Senator Feingold’s suites, with the exception of one responder who was in the hallway adjacent to Senator Daschle’s office on the 6th floor but did not enter either suite. All 18 persons (including 5 first responders) in Senator Daschle’s 6th-floor suite had positive nasal cultures; a much lower proportion had positive nasal swabs on the 5th-floor Daschle suite (28%) and 6th-floor Feingold suite (13%).

**Table 3:** Proportion of persons with positive nasal swabs for *Bacillus anthracis* in the immediate exposure area, by office and floor,^a^ Capitol Hill, Washington, D.C.

Floor	Senate office	Persons in area	Positive nasal swabs (% positive)
6	Daschle	18	18 (100)
	Feingold	15	2 (13)
5	Daschle	25	7 (28)
	Feingold	12	0 (0)
Total		70	27 (39)

^a^One responder with a positive nasal swab who was in the 6th-floor hallway did not enter the Daschle or Feingold suites and was not included in this table.

 Repeat nasal swabs from the 28 persons with initially positive nasal cultures for *B. anthracis* were negative for all persons at 7 days postexposure. Serologic tests were negative for anti-PA IgG antibodies in all persons at 7, 21, and 42 days after exposure. To date, anthrax has not developed in anyone in this cohort or in the larger cohort of persons on Capitol Hill.

## Discussion

 Among the series of bioterrorism incidents during 2001 related to *B. anthracis*–contaminated envelopes, this event was unique because it was the first with a known source of exposure, enabling a rapid public health response by a multidisciplinary team including law enforcement officers, medical and public health personnel, laboratory personnel, industrial hygienists, and engineers. The known source enabled us to assess the usefulness of nasal swab cultures in determining exposure to *B. anthracis*.

 The contaminated letter purportedly contained about 2 g of powder, with each gram reported to contain between 100 billion to 1 trillion spores (14). The recovery of *B. anthracis* from nasal cultures was limited to persons who were inside Senator Daschle or Feingold’s offices or in the hallway joining the two offices. Nasal swab results suggest that the ventilation system played a very small role, if any, in the spread of anthrax spores in HSOB. Based on proportions of persons with positive nasal swabs, most dissemination likely occurred through room currents from the 6th to the 5th floor of the Daschle suite via an open staircase; closed doors that blocked air currents were most likely the reason a smaller proportion in Senator Feingold’s office had positive nasal cultures despite being adjacent to Senator Daschle’s office.

 Swabs were taken within 1 day of the initial event from all 71 persons in the immediate exposure area, including those with positive nasal cultures for *B. anthracis.* However, in others with negative results, testing was not done for up to 4 days. Although these persons were located outside the immediate exposure area, it is uncertain whether prompt antibiotic administration, a delay in nasal swab testing, or both, may have had an effect on those nasal culture results. In one animal model involving macaques, large inhaled doses of anthrax spores in a controlled setting yielded *B. anthracis* in nasal swabs of all animals within 24 hours of exposure, and although sensitivity decreased as time progressed, positive nasal cultures were recovered in some macaques 1 week after exposure (15). In the Florida anthrax investigation, positive nasal cultures were detected in a person >1 week after presumed exposure (5). Repeat swabs from the persons with initially positive cultures were negative at 7 days postexposure, but prophylaxis administration may have influenced those results. The greatest sensitivity for recovery of *B. anthracis* can be achieved by obtaining nasal swab specimens as early as possible after recognized exposure.

 Nasal swabs served as an epidemiologic tool; we considered the work locations of those with positive nasal swabs to be areas at risk for anthrax exposure. However, interpretation of positive or negative nasal swab results for individual risk assessment of anthrax disease has not been evaluated, and nasal swabs should not be used for this purpose. In the case of one person who died after exposure to anthrax, a nasal swab culture was negative (16). Likewise, environmental sampling may be a valuable component of assessment of areas of risk, but individual environmental samples are not sufficient to determine a person's risk for anthrax.

 Two other issues deserve mention. First, the use of PPE may be an effective barrier to exposure to *B. anthracis* spores, although its efficacy could not be addressed in this investigation; no responder entering Senator Daschle’s office wore PPE before entering the office, and all had positive nasal swabs. Second, while subclinical anthrax infection has been documented in persons with continuous exposure to *B. anthracis* spores (9), the lack of serologic conversion in persons with positive nasal cultures suggests that no apparent asymptomatic infection occurred during this event, when prophylaxis was promptly initiated and continued.

 Since the initial events of October 15, more information has become available—four cases of inhalational anthrax, two of them fatal, occurred in USPS employees from the Washington, D.C., Postal Distribution Center where Senator Daschle’s envelope was sorted (7,12,16), and a fifth case occurred in an employee of another mail facility, which receives government mail from the Washington, D.C., Distribution Center. These events led to new recommendations to expand the traceback for future events through the entire path to envelope origin. In addition, updated prophylaxis and treatment protocols, including options for vaccination, and subsequent recommendations for a comprehensive response to a bioterrorism attack involving *B. anthracis* have been published (17–21). In Table 4, specific recommendations are given for a comprehensive public health response and epidemiologic investigation that prevent further spread, identify and treat those at risk, and avoid mass administration of prolonged prophylaxis to persons not considered at risk for anthrax in the event of a future bioterrorist attack.

**Table 4:** Recommendations for public health response to, and epidemiologic assessment of, the opening of an envelope suspected of containing *Bacillus anthracis* spores

Proper training on handling suspicious envelopes and packages
Use of personal protective equipment
Rapid identification of *B. anthracis* spore
Shutdown of ventilation system
Evacuation of immediate and surrounding area
Prompt administration of antimicrobial prophylaxis, in conjunction with offering vaccine under appropriate circumstances, to persons in immediate area
Use of epidemiologic tools to define exposure area and assess risk in the surrounding area
Nasal cultures and environmental samples for *B. anthracis*
Floor diagrams
Building ventilation
Traceback of letter path from destination to origin

 In conclusion, a rapid and coordinated public health response helped avert an anthrax outbreak by identifying and administering prophylaxis to persons at high risk for disease. Nasal swabs can provide useful information about the extent of exposure to *B. anthracis* spores to assist with defining groups at risk.

Epidemiologic assessment of risk for anthrax in persons in settings affected by a biological attack is complex, and much remains to be learned. In the meantime, a well-developed public health infrastructure, effective antimicrobial prophylaxis strategies, and effective guidelines for management based on past experiences are essential in our defense against future bioterrorism events.

## References

[1] Centers for Disease Control and Prevention. Bioterrorism alleging use of anthrax and interim guidelines for management—United States, 1998. MMWR Morb Mortal Wkly Rep. 1999;48:69–74. 10023627

[2] Inglesby TV, Henderson DA, Bartlett JG, Ascher MS, Eitzen E, Friedlander AM, Anthrax as a biological weapon. JAMA. 1999;281:1735–45. 10.1001/jama.281.18.1735 10328075

[3] Centers for Disease Control and Prevention. Biological and chemical terrorism: Strategic plan for preparedness and response. MMWR Morb Mortal Wkly Rep. 2000;49(RR-4). 10803503

[4] Bush LM, Abrams BH, Beall A, Johnson CC. Index case of fatal inhalational anthrax due to bioterrorism in the United States. N Engl J Med. 2001;345:1607–10. 10.1056/NEJMoa012948 11704685

[5] Traeger MS, Wiersma S, Rosenstein NE, Malecki J, Shepard C, Raghunathan P, First case of bioterrorism-related anthrax, United States, Palm Beach County, Florida, 2001. Emerg Infect Dis. 2002;8:1029–34. 12396910 10.3201/eid0810.020354 PMC2730309

[6] Freedman A, Afonja O, Chang MW, Mostashari F, Blaser M, Perez-Perez G, Cutaneous anthrax associated with microangiopathic hemolytic anemia and coagulopathy in a 7-month-old infant. JAMA. 2002;287:869–74. 10.1001/jama.287.7.869 11851579

[7] Jernigan JA, Stephens DS, Ashford DA, Omenaca C, Topiel MS, Galbraith M, Bioterrorism-related inhalational anthrax: the first 10 cases reported in the United States. Emerg Infect Dis. 2001;7:933–44. 11747719 10.3201/eid0706.010604 PMC2631903

[8] Carr EA, Rew RR. Recovery of *Bacillus anthracis* from the nose and throat of apparently healthy workers. J Infect Dis. 1957;100:169–71. 13416651 10.1093/infdis/100.2.169

[9] Norman PS, Ray GR, Brachman PS, Plotkin SA, Pagano JS. Serologic testing for anthrax antibodies in workers in a goat hair processing mill. Am J Hyg. 1960;71:32–7. 10.1093/oxfordjournals.aje.a120132 14427621

[10] Sirisanthana T, Nelson KE, Ezzell JW, Abshire TG. Serological studies of patients with cutaneous and oral-oropharyngeal anthrax from northern Thailand. Am J Trop Med Hyg. 1988;39:575–81. 3144920 10.4269/ajtmh.1988.39.575

[11] Harrison LH, Ezzell JW, Abshire TG, Kidd S, Kaufmann AF. Evaluation of serologic tests for diagnosis of anthrax after an outbreak of cutaneous anthrax in Paraguay. J Infect Dis. 1989;160:706–10. 2507648 10.1093/infdis/160.4.706

[12] Dewan PK, Fry AM, Laserson K, Tierney BC, Quinn CP, Hayslett JA, Inhalational anthrax outbreak among postal workers, Washington, D.C., 2001. Emerg Infect Dis. 2002;8:1066–72. 12396917 10.3201/eid0810.020330 PMC2730301

[13] Centers for Disease Control and Prevention. Evaluation of *Bacillus anthracis* contamination inside the Brentwood mail processing and distribution center—District of Columbia, October 2001. MMWR Morb Mortal Wkly Rep. 2001;50:1129–33. 11824387

[14] Kennedy H. Daschle letter bombshell billions of anthrax spores. New York Daily News. October 31, 2001:5. Available from: URL: http://www.nydailynews.com

[15] Hail AS, Rossi CA, Ludwig GV, Ivins BE, Tammariello RF, Henchal EA. Comparison of noninvasive sampling sites for early detection of *Bacillus anthracis* spores from Rhesus monkeys after aerosol exposure. Mil Med. 1999;164:833–7. 10628152

[16] Borio L, Frank D, Mani V, Chiriboga C, Pollanen M, Ripple M, Death due to bioterrorism-related inhalational anthrax. JAMA. 2001;286:2554–9. 10.1001/jama.286.20.2554 11722269

[17] Centers for Disease Control and Prevention. Update: Investigation of bioterrorism-related anthrax and interim guidelines for exposure management and antimicrobial therapy, October, 2001. MMWR Morb Mortal Wkly Rep. 2001;50:909–19. 11699843

[18] Centers for Disease Control and Prevention. Updated recommendations for antimicrobial prophylaxis among asymptomatic pregnant women after exposure to *Bacillus anthracis.* MMWR Morb Mortal Wkly Rep. 2001;50:960. 11708594

[19] Centers for Disease Control and Prevention. Update: Interim recommendations for antimicrobial prophylaxis for children and breastfeeding mothers and treatment of children with anthrax. MMWR Morb Mortal Wkly Rep. 2001;50:1014–6. 11724160

[20] Centers for Disease Control and Prevention. Additional options for preventive treatment for persons exposed to inhalational anthrax. MMWR Morb Mortal Wkly Rep 2001;50:1142, 1151.

[21] Inglesby TV, O’Toole T, Henderson DA, Bartlett JG, Ascher MS, Eitzen E, Anthrax as a biological weapon, 2002. JAMA. 2002;287:2236–52. 10.1001/jama.287.17.2236 11980524

